# Understanding the inter‐relationships of type 2 diabetes and hypertension with brain and cognitive health: A UK Biobank study

**DOI:** 10.1111/dom.14658

**Published:** 2022-02-24

**Authors:** Danielle Newby, Victoria Garfield

**Affiliations:** ^1^ Department of Psychiatry University of Oxford, Warneford Hospital Oxford UK; ^2^ MRC Unit for Lifelong Health and Ageing at UCL, Department of Population Science and Experimental Medicine, Institute of Cardiovascular Science University College London London UK

**Keywords:** brain health, cardiovascular disease, dementia, diabetes, epidemiology, hypertension

## Abstract

**Aim:**

To understand the impact of diabetes and co‐morbid hypertension on cognitive and brain health.

**Materials and Methods:**

We used data from the UK Biobank cohort consisting of ~500 000 individuals aged 40 to 69 years. Our outcomes included brain structural magnetic resonance imaging variables and cognitive function tests in a maximum of 38 918 individuals. We firstly tested associations with all outcomes between those with diabetes (*n* = 2043) and without (*n* = 36 875) and, secondly, compared those with co‐morbid diabetes/hypertension (*n* = 1283) with those with only diabetes (*n* = 760), hypertension (*n* = 9649) and neither disease (*n* = 27 226). Our analytical approach comprised linear regression models, with adjustment for a range of demographic and health factors. Standardized betas are reported.

**Results:**

Those with diabetes had worse brain and cognitive health for the majority of neuroimaging and cognitive measures, with the exception of g fractional anisotropy (white matter integrity), amygdala, pairs matching and tower rearranging. Compared with individuals with co‐morbid diabetes and hypertension, those with only hypertension had better brain health overall, with the largest difference observed in the pallidum (*β* = .189, 95% CI = 0.241; 0.137), while those with only diabetes differed in total grey volume (*β* = .150, 95% CI = 0.122; 0.179). Individuals with only diabetes had better verbal and numeric reasoning (*β* = .129, 95% CI = 0.077; 0.261), whereas those with only hypertension performed better on the symbol‐digit substitution task (*β* = .117, 95% CI = 0.048; 0.186).

**Conclusions:**

Individuals with co‐morbid diabetes and hypertension have worse brain and cognitive health compared with those with only one of these diseases. These findings potentially suggest that prevention of both diabetes and hypertension may delay changes in brain structure, as well as cognitive decline and dementia diagnosis.

## 1 INTRODUCTION

Type 2 diabetes is associated with an excess risk of dementia,^1^, ^2^ worse cognitive function^3^, ^4^ and changes in brain structure and function.^1^ Large‐scale population‐based studies, such as the UK Biobank (UKB), have recently expanded our understanding of the relationship between diabetes, cognitive function and brain health.^5^, ^6^, ^7^ Important insights from these studies include that diabetes is associated with slower reaction times,^5^, ^7^ poorer brain health as indexed by structural and diffusion indices, and an increased risk of vascular, Alzheimer's disease and all‐cause dementia.^6^, ^7^ The relationship between hypertension and a range of adverse cognitive and brain health variables is also well established.^8^, ^9^, ^10^, ^11^, ^12^ Hypertension in mid‐life is also known to increase the risk of both Alzheimer's disease and vascular dementia.^10^, ^12^

Importantly, global estimates show that more than 50% of adults with diabetes also have a hypertension diagnosis.^13^ Epidemiological data suggest that those with both diabetes and hypertension have a 23% excess risk of dementia, in comparison with individuals with diabetes but no hypertension whose excess risk is around 19%. Also, evidence from the baseline UKB sample suggests that the additive combination of diabetes and hypertension is related to worse cognitive performance when accounting for a number of confounding factors.^5^

As described above, there have been recent important advances in how diabetes and hypertension are associated with cognitive and brain health, yet a number of evidence gaps remain to be filled. Despite the fact that over half of adults with diabetes also have hypertension and that this is associated with an excess dementia risk, no research has investigated whether co‐morbid diabetes and hypertension is associated with worse brain health, using structural neuroimaging outcomes; nor have they used a breadth of measures to define diabetes in this context. In this study, we used data from the UKB to test two a priori hypotheses: (a) participants with type 2 diabetes (referred to as diabetes from here on) have poorer brain health and cognitive function compared with those without diabetes; and (b) participants with diagnosed diabetes and hypertension have poorer brain health and cognitive function than those with only one of these diseases or with neither of these diseases. We tested both of our hypotheses using multiple neuroimaging outcomes and cognitive tests to capture brain health and cognitive function.

## 2 MATERIALS AND METHODS

### 2.1 Study design

A cross‐sectional study of 38 918 participants with brain magnetic resonance imaging (MRI) data in the UKB was used to determine the association between diabetes and those with either hypertension and/or diabetes with several global brain volumes, grey subcortical, white matter micro and macrostructures volumes and cognitive tests.

### 2.2 Setting

The UKB is a large prospective cohort of more than half a million participants. All participants, aged 40‐69 years, initially attended baseline assessment visits from 2006 to 2010 where they completed a series of physical, sociodemographic, cognitive and medical assessments.^14^ Follow‐up visits have taken place, including typical assessments as with the baseline visit but with whole body imaging including MRI brain imaging. To date, nearly 50 000 participants have had MRI brain imaging carried out with more than 40 000 participants with data currently available. These participants also completed the same battery of assessments as at the baseline visit. The UKB received ethical approval from the Research Ethics Committee (11/NW/0382). Volunteers gave informed consent for their participation.

### 2.3 Participants

Participants who attended the assessment centre for an MRI brain scan were included in this study. These participants also provided demographic, health, and socioeconomic information using touchscreen questionnaires as well as taking part in a nurse‐led interview asking questions about medical history and medications. Participants who reported they had any neurodegenerative or related diseases were excluded from this analysis (*n* = 612) as in previous work.^11^ A full list of these diseases and UKB field codes for all the variables used in this work can be found in Tables S1 and S2. We additionally removed participants with any diagnosis of either type 1 (*n* = 152), gestational diabetes (*n* = 4) or unspecified diabetes (*n* = 6) after defining our diabetes population. This resulted in 38 918 participants included in our analysis.

### 2.4 Variables

#### 2.4.1 Exposures: diabetes and hypertension

Participants were defined as having diabetes using information on self‐report (diagnosis/medications) (*n* = 1925), biochemistry (*n* = 586) and clinical data (*n* = 1366), with 68% (*n* = 1390) overlapping with two or more diabetes phenotypes (Figure S1). More specifically, ‘known diabetes’ was defined as those captured via self‐report and clinical data, while those captured via biochemistry (high HbA1c) were classified as having ‘undiagnosed diabetes’ (see the supporting information). However, for ease we refer to all of these cases as ‘diabetes’ throughout this paper. Participants were defined as having hypertension based on self‐reported diagnosis and self‐reported medications. The UKB field codes to define diabetes and hypertension used in this work and further information regarding the definitions of phenotypes can be found in the supporting information.

#### 2.4.2 Outcome: neuroimaging

Brain MR images were acquired on a Siemens Skyra 3‐T scanner with a standard Siemens 32‐channel head coil. We utilized imaging‐derived phenotypes derived from the raw brain MRI images that were generated using an image‐processing pipeline developed and quality controlled centrally by the UKB.^15^ In this work, we included total brain volume, grey matter volume, white matter hyperintensity (WMH) volume and ventricular cerebrospinal fluid (CSF). We also analysed subcortical volumes (accumbens, amygdala, caudate, hippocampus, pallidum, putamen and thalamus) by averaging left and right measures and latent measures of tract‐averaged fractional anisotropy (FA) and mean diffusivity (MD) of all the WM tracts. FA and MD are two metrics imaged with diffusion‐tensor imaging that are indicative of WM tract microstructural integrity: higher FA values suggest better health, whereas higher MD suggests worse WM tract health. Because of the high correlation of the WM microstructural properties across the brain of individual regions of FA and MD, single general latent measures of FA (gFA) and MD (gMD) were created using confirmatory factor analysis, as described previously.^6^, ^16^ Because of the low correlation of the subcortical volumes (Figure S2) we did not create latent measures for these brain measures. Outlier datapoints, defined as being further than ± 4 SD from the mean, were excluded (< 1% of values).

#### 2.4.3 Outcome: cognitive function

Cognitive function was assessed using the cognitive tests verbal numerical reasoning, pairs matching (memory), reaction time (processing speed), matrix pattern (non‐verbal reasoning), symbol‐digit substitution (processing speed/executive function), tower rearranging (executive function/planning), and the difference between trail‐making test (TMT) B and A (processing speed/executive function). Further information regarding the cognitive tests can be found at https://biobank.ctsu.ox.ac.uk/crystal/label.cgi?id=100026. Verbal numerical reasoning, pairs matching and reaction time cognitive tests were bespoke to the UKB. The remaining cognitive tests are validated tests, which were additionally administered at the imaging visits from 2016 (the imaging visit follow‐ups began in 2014). Higher values indicate better cognitive performance on verbal and numeric reasoning, matrix reasoning, symbol‐digit substitution and tower rearranging, and worse cognitive performance on the reaction time pairs matching and TMT B − TMT A.

#### 2.4.4 Covariates

In this study we selected the following covariates for adjustment in our models: demographic measures (age + sex + deprivation + educational attainment + ethnicity), as well as head size and MRI scanner position variables (for neuroimaging outcomes only), and standard cardiovascular risk factor measures (smoking + body mass index [BMI] + hypertension + high cholesterol).

Age at assessment was measured in whole years and sex was self‐reported as male or female. Educational qualifications were self‐reported and were dichotomized into whether participants held a university/college degree or not. Self‐reported ethnicity was dichotomized into White or non‐White and if was missing was obtained from the baseline assessment visit. Assessment centre was a multilevel variable of the assessment centres utilized for the repeated imaging visits (*n* = 3). Townsend deprivation index was calculated before the baseline visit and was split into quintiles. BMI was calculated from valid height (cm) and weight (kg) measurements obtained by the UKB and was used as a continuous measure. Smoking status was self‐reported and categorized into never smoked, current or former smoker. For hyperlipidaemia, a combination of self‐reported and medication records were used. Where participants responded ‘Do not know’ or ‘Prefer not to answer’ these were treated as missing (< 1%) and missing data were not imputed.

### 2.5 Statistical methods

All analyses were performed using R version 4.0.2. Descriptive statistics were generated to characterize the study cohort at baseline.

#### 2.5.1 Modelling approach

Natural log transformations were applied to reaction time and WMH. For the pairs matching cognitive test a natural log transformation +1 (ln[x + 1]) was used and, for TMT B − A, a square root transformation was applied to make these outcomes normally distributed prior to analysis. All other outcome variables were normally distributed. Standardized beta coefficients are reported for all analyses to facilitate comparison of associations across the neuroimaging and cognitive outcomes. We converted all of our betas so that they would be in the same direction to aid interpretability. Therefore, all standardized betas with a positive value indicate better brain health or cognitive performance, and negative values indicate poorer brain health and cognitive performance. For the neuroimaging outcomes, WMH, vascular CSF, and gMD results were converted to the same direction as all other neuroimaging measures and, for the cognitive outcomes, reaction time, pairs matching and TMT B − A results were converted to the same direction as all the other cognitive tests.

In stage 1 of our analyses we tested our first a priori hypothesis and compared brain and cognitive function measures between those with (*n* = 2043) and without diabetes (*n* = 36 875). In stage 2, we tested our second a priori hypothesis that co‐morbid diabetes and hypertension would associate more strongly with worse brain and cognitive health. Thus, we compared outcomes across those with both hypertension and diabetes (*n* = 1283) versus those with only diabetes (*n* = 760), only hypertension (*n* = 9649) or no hypertension/diabetes (*n* = 27 226). We fitted two linear regression models for stages 1 and 2 of our analyses; the first was adjusted for demographics (model 1) and the second was additionally adjusted for cardiovascular‐related covariates, as described earlier (model 2).

## 3 RESULTS

### 3.1 Sample characteristics

A total of 38 918 individuals were included in the study, of whom 36 875 did not have diabetes and 2043 had known diabetes. The age range of participants in this imaging subset used in this work was 44‐82 (mean 63.62, SD 7.55) years. Those with diabetes were more probably male, to not have a degree, not be of Europid ethnicity, be current smokers, reside in the most deprived quintile, have a higher BMI, and have the highest prevalence of hypercholesterolaemia and hypertension (Table 1).

**TABLE 1:** Characteristics of the UK Biobank participants at imaging visit stratified by diabetes diagnosis

Description	No diabetes (*n* = 36 875)	Diabetes (*n* = 2043)	*N*
Age, y (mean [SD])	63.5 (7.55)	65.8 (7.17)	38 918
Sex (male *N* [%])	17 030 (46.2)	1308 (64.0)	38 918
Ethnicity (White *N* [%])	35 778 (97.3)	1875 (92.1)	38 815
Education (degree *N* [%])	18 072 (49.5)	784 (38.9)	38 525
Townsend deprivation (*N* [%])			
1	7454 (20.2)	335 (16.4)	38 883
2	7427 (20.2)	334 (16.4)	
3	7378 (20.0)	407 (19.9)	
4	7343 (19.9)	430 (21.1)	
5	7240 (19.7)	535 (26.2)	
Assessment centre (*N* [%])			
Cheadle	22 928 (62.2)	1282 (62.8)	38 918
Reading	4770 (12.9)	252 (12.3)	
Newcastle	9177 (24.9)	509 (24.9)	
BMI, kg/m^2^ (mean [SD])	26.3 (4.27)	29.7 (5.18)	37 626
Smoking status (*N* [%])			
Non‐smoker	23 098 (63.2)	1092 (54.1)	38 541
Previous	1264 (3.46)	77 (3.81)	
Current	12 159 (33.3)	851 (42.1)	
Hypercholesterolaemia (*N* [%])	8065 (21.9)	1424 (69.7)	38 918
Hypertension (*N* [%])	9649 (26.2)	1283 (62.8)	38 918
Total brain volume, mm^3^ (mean [SD])	1 162 216 (111 176)	1 149 440 (109 986)	38 908
Grey matter, mm^3^ (mean [SD])	616 074 (55 455)	601 073 (56 175)	38 911
WMH, mm^3^ (median [IQR])	2708 (3936)	4058 (6175)	37 168
gFA units M (SD)	0.01 (0.55)	−0.10 (0.58)	34 718
gMD units M (SD)	−0.01 (0.46)	0.12 (0.50)	34 718
Ventricular CSF, mm^3^ (mean [SD])	35 817 (15 847)	41 925 (17 708)	38 721
Hippocampus, mm^3^ (mean [SD])	3843 (433)	3755 (446)	38 876
Accumbens, mm^3^ (mean [SD])	443 (105)	406 (103)	38 902
Amygdala, mm^3^ (mean [SD])	1245 (216)	1247 (218)	38 896
Pallidum, mm^3^ (mean [SD])	1779 (221)	1727 (233)	38 835
Putamen, mm^3^ (mean [SD])	4801 (569)	4694 (571)	38 872
Caudate, mm^3^ (mean [SD])	3473 (419)	3421 (419)	38 870
Thalamus, mm^3^ (mean [SD])	7667 (727)	7465 (729)	38 851
Pairs matching—incorrect matches (mean [SD])	3.65 (2.86)	3.82 (3.09)	35 898
Verbal and numerical reasoning—correct answers (mean [SD])	6.65 (2.05)	6.27 (2.13)	35 837
Reaction time, s (median [IQR])	574 (129)	593 (137)	36 323
Trail‐making test B − A, s (median [IQR])	292 (197)	326 (235)	24 402
Matrix reasoning—correct answers (mean [SD])	8.02 (2.12)	7.53 (2.28)	25 286
Symbol‐digit substitution—correct answers (mean [SD])	19.1 (5.23)	17.2 (5.36)	25 320
Tower rearranging—correct answers (mean [SD])	9.93 (3.22)	9.54 (3.37)	25 074

*Note*: Townsend deprivation is split into quintiles, where 5 is most deprived. For the brain measures, larger values for WMHs, ventricular CSF and gMD indicate poorer brain health, whereas for all other brain measures smaller values indicate poorer brain health. For the cognitive tests, lower values indicate poorer cognition for verbal and numerical reasoning, matrix reasoning, symbol‐digit substitution and tower arranging, and for pairs matching, reaction time and trail‐making test B – A, higher values indicate poorer cognition.

Abbreviations: BMI, body mass index; CSF, cerebrospinal fluid; gFA, g fractional anisotropy; gMD, g mean diffusivity; IQR, interquartile range; WMH, white matter hyperintensity.

The demographics of participants stratified by diabetes and hypertension diagnosis can be found in Table S3. Those with both diabetes and hypertension (*n* = 1283) were more probably male, without a degree, have higher BMI, be ex‐smokers and have high cholesterol. Those with just diabetes (*n* = 760) were younger and more probably not of Caucasian ethnicity and were more probably current smokers. Those with hypertension only (*n* = 9649) tended to have slightly fewer or comparable co‐morbidities compared with those with diabetes or with both diseases, but had a higher prevalence compared with those with no diabetes or hypertension.

### 3.2 Association between diagnosed diabetes and neuroimaging and cognitive outcomes

**FIGURE 1:**
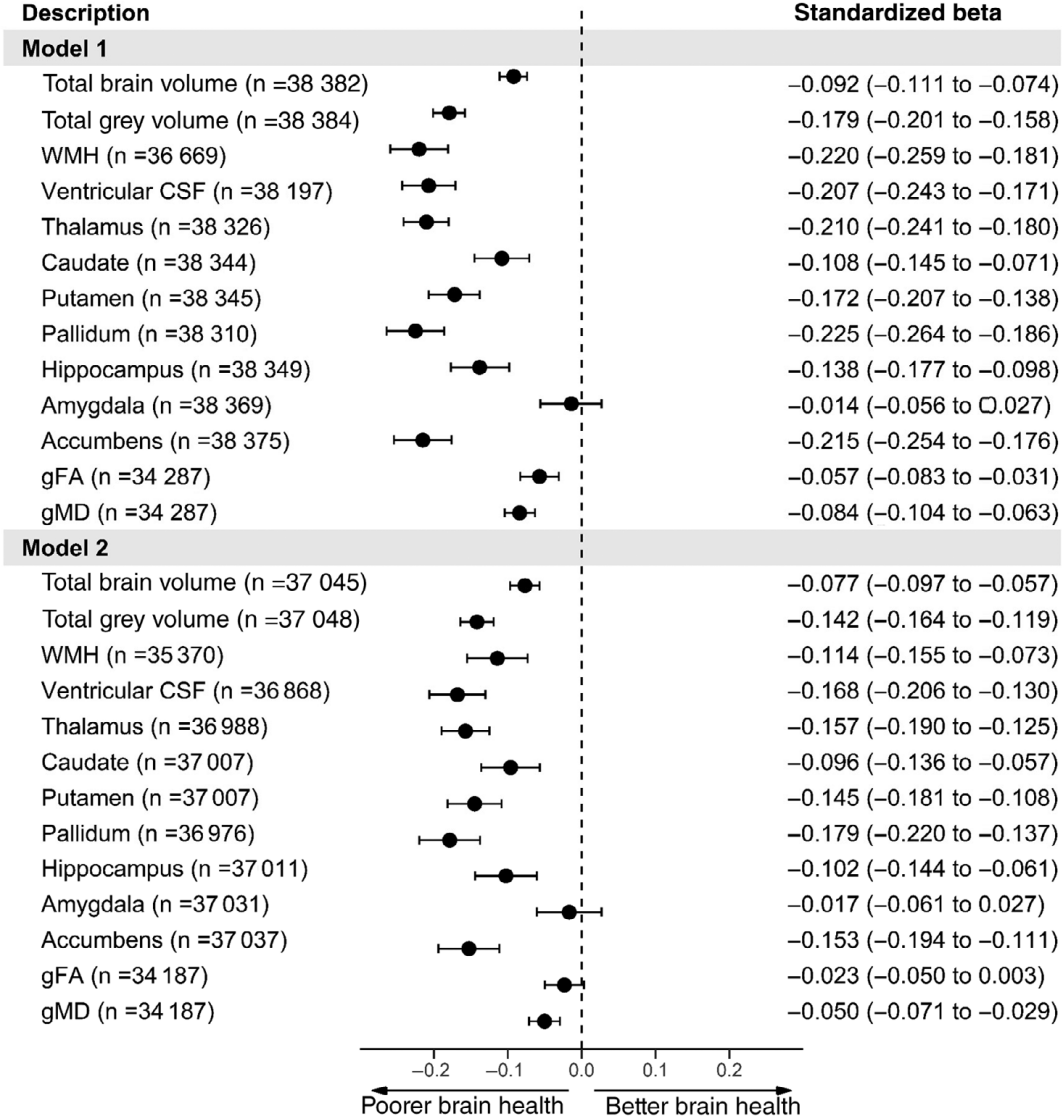
Forest plot of the association between diabetes (*n* = 2043 cases, *n* = 36 875 controls) and neuroimaging outcomes. Model 1 = adjusted for age + sex + deprivation + ethnicity + educational attainment + head size + scanner position variables. Model 2 = Model 1 + body mass index + CVD + hypercholesterolaemia + hypertension + smoking. WMH, ventricular CSF and gMD results were converted to the same direction of all other brain measures so that higher values indicate better brain health compared with reference level, for ease of comparisons. CSF, cerebrospinal fluid; CVD, cardiovascular disease; gFA, g fractional anisotropy; gMD, g mean diffusivity; WMH, white matter hyperintensity

Individuals with diabetes are associated with poorer brain measures compared with those without diabetes, with the exception of the amygdala subcortical region (Figure 1, model 1). However, when adjusting for cardiovascular‐related variables, associations were attenuated with gFA, a measure of WM integrity, no longer different between those with and without diabetes (Figure 1, model 2). We also present the standardized betas of diabetes and hypertension from model 2 in the supporting information for comparison of these effects as single covariates within our model (Table S4).

**FIGURE 2:**
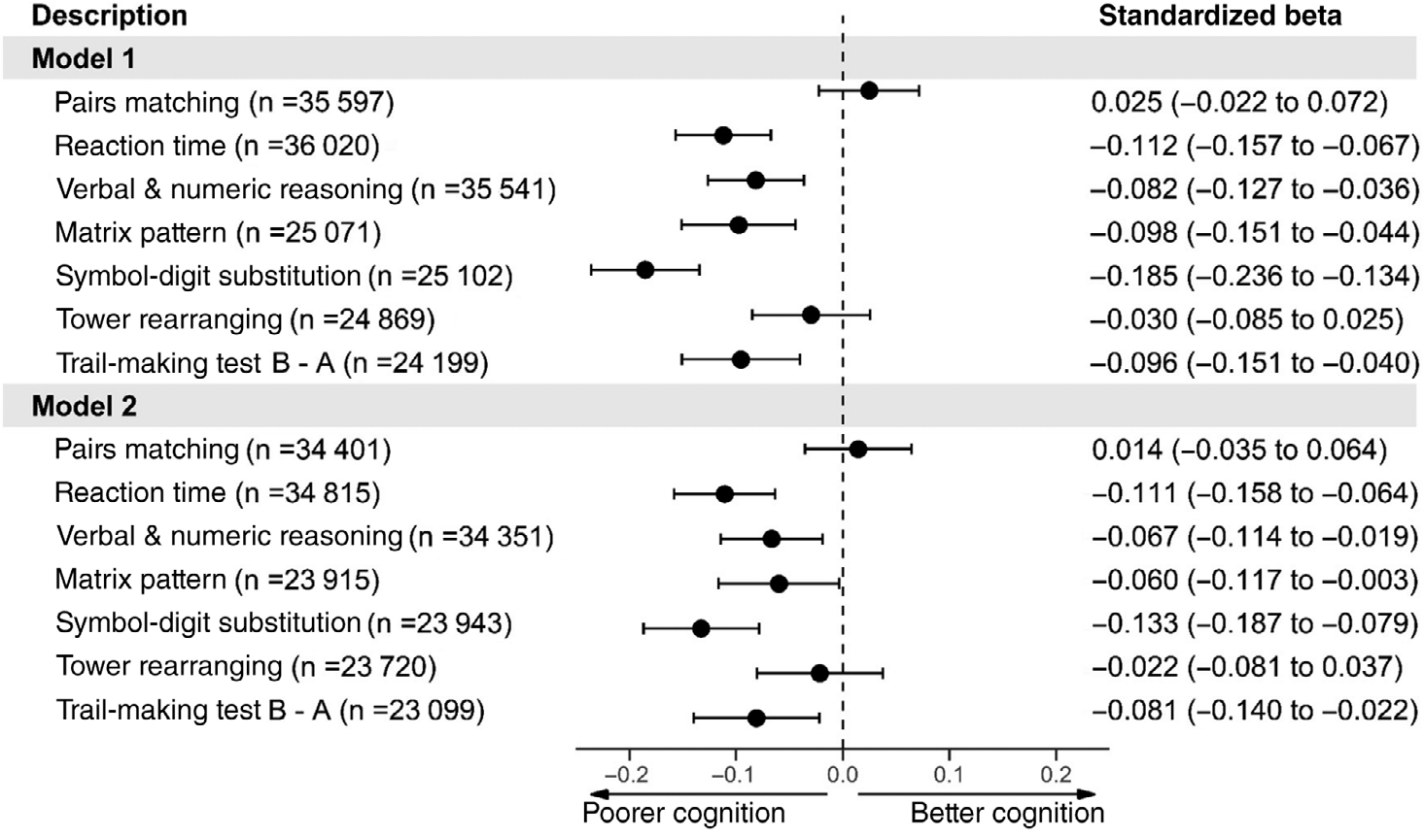
Forest plot of the association between diabetes (*n* = 2043 cases, *n* = 36 875 controls) and cognitive function. Model 1 = adjusted for age + sex + deprivation + ethnicity + educational attainment. Model 2 = Model 1 + body mass index + CVD + hypercholesterolaemia + hypertension + smoking. Reaction time, pairs matching and trail‐making test B − A results were converted to the same direction of all other cognitive tests so that higher values indicate better cognitive performance and lower values indicate poorer cognitive performance compared with controls. CVD, cardiovascular disease

When we compared individuals with and without diabetes using the cognitive tests, we found that in the partially adjusted models (model 1) those with diabetes had slower reaction times, were slower on the TMT, had poorer verbal and numeric reasoning and performed worse on matrix pattern and symbol‐digit substitution. There were no differences between groups for the pairs matching or tower rearranging (Figure 2, model 1). Upon further adjustment for cardiovascular‐related covariates (model 2), results were again attenuated, particularly for the matrix pattern cognitive and symbol‐digit substitution tests when comparing standardized betas between models 1 and 2 (Figure 2, model 2). Again, the standardized betas of diabetes and hypertension from model 2 are presented in the supporting information for comparison of these effects as single covariates in our model (Table S5).

We carried out an additional sensitivity analysis removing participants with low BMI (< 18.5 kg/m^2^) as this could be a proxy for frailty. Removal of participants with low BMI did not change our results (supporting information S3‐S4).

### 3.3 Association between co‐morbid diabetes and hypertension versus only diabetes/hypertension versus neither: neuroimaging and cognitive outcomes

To investigate the inter‐relationship between co‐morbid diabetes and hypertension and neuroimaging and cognitive outcomes, we further split our sample and created four groups, based on both diabetes and hypertension diagnosis. Using those individuals with both diabetes and hypertension as the reference group allowed the comparison of neuroimaging and cognitive outcomes with individuals with either diabetes or hypertension or neither disease to understand how having both diseases was associated with brain and cognitive health.

**FIGURE 3:**
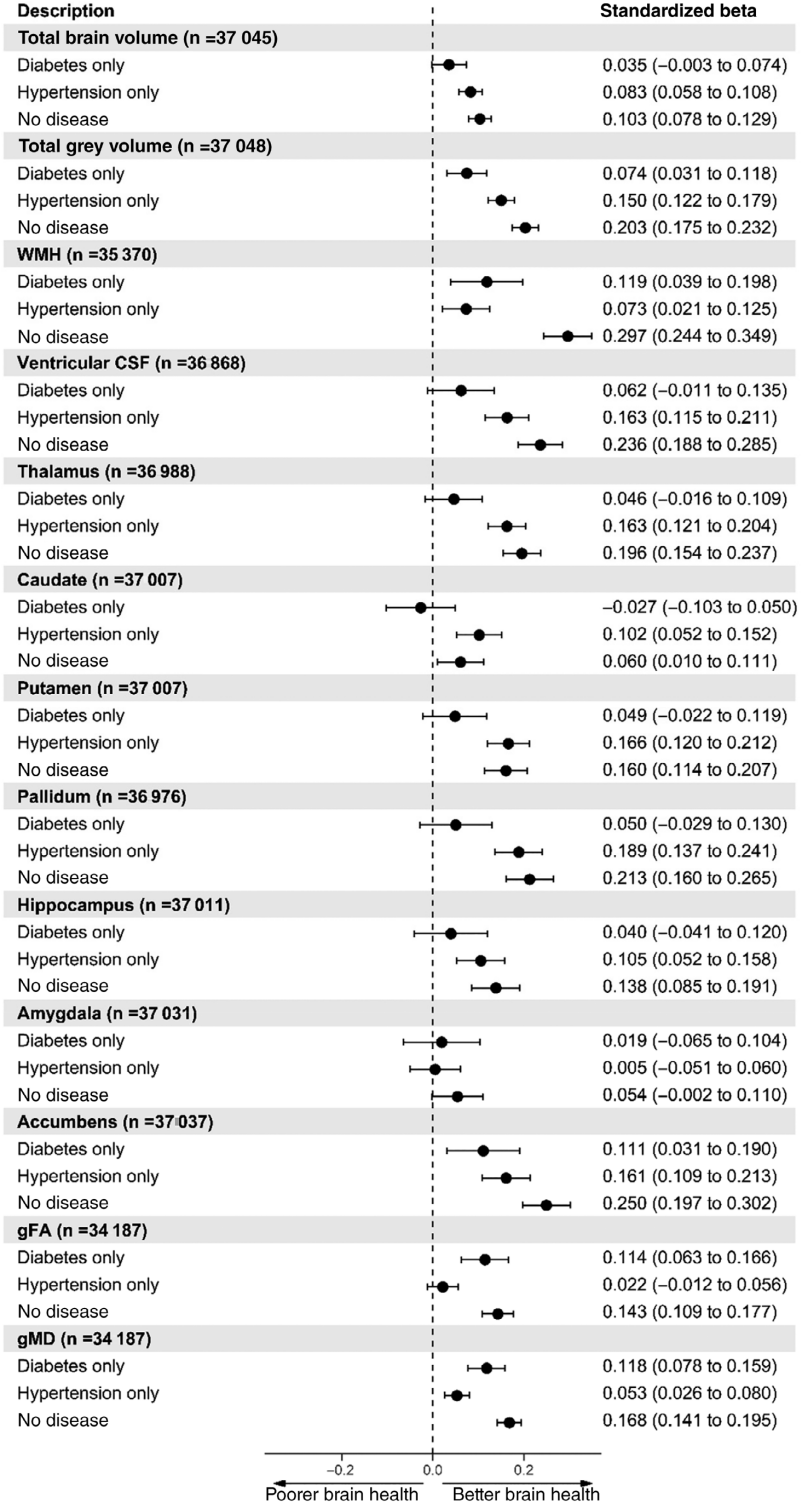
Forest plot of the association between disease status (both diabetes and hypertension [*n* = 1283], diabetes only [*n* = 760], hypertension only [*n* = 9649] and no diabetes and hypertension [*n* = 27 226]) and neuroimaging outcomes. Individuals with both diabetes and hypertension were set as the reference level. Model 2 = adjusted for age + sex + deprivation + ethnicity + educational attainment + head size + scanner position variables + body mass index + CVD + hypercholesterolaemia + smoking. WMH, ventricular CSF and gMD results were converted to the same direction of all other brain measures so that higher values indicate better brain health compared with reference level, for ease of comparisons. CSF, cerebrospinal fluid; CVD, cardiovascular disease; gFA, g fractional anisotropy; gMD, g mean diffusivity; WMH, white matter hyperintensity

Figure 3 shows the fully adjusted (model 2) results of comparing individuals with co‐morbid diabetes and hypertension with those with either hypertension or diabetes and those not with these diseases with neuroimaging outcomes. The results for model 1, only adjusted for demographics, can be found in Figure S5. From Figure 3, for the vast majority of neuroimaging outcomes, those with hypertension had better brain measures (i.e. larger brain volumes, smaller WMH) compared with those with both diabetes and hypertension, apart from amygdala and gFA. Individuals with diabetes had larger total grey matter but there were no differences between individuals with diabetes only versus those with both hypertension and diabetes for total brain volume and subcortical volumes, apart from the accumbens. Those with diabetes had lower WMH and better WM integrity measures (gFA and gMD). Apart from the amygdala, those with neither disease had better brain health compared with those with both diseases.

**FIGURE 4:**
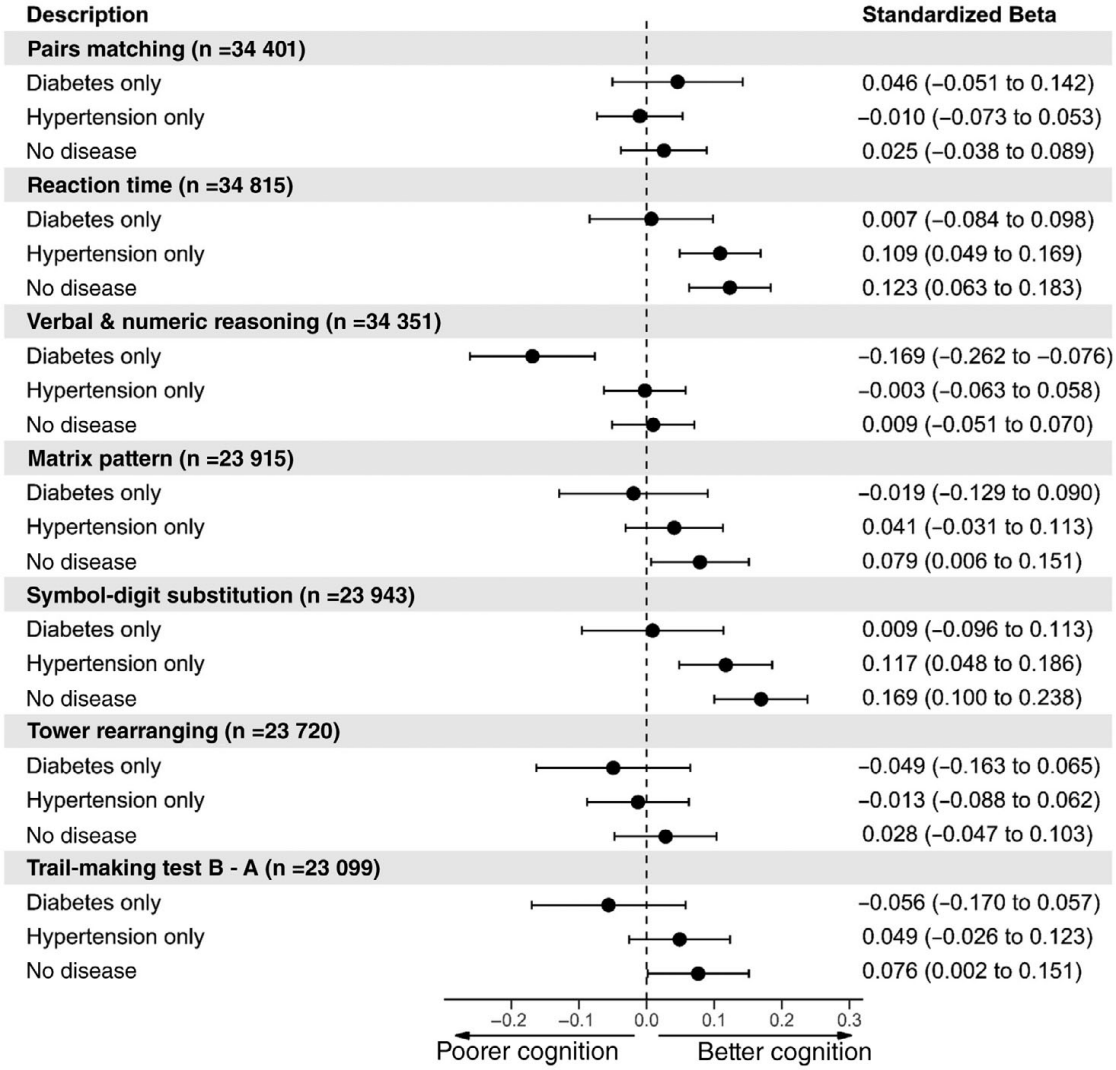
Forest plot of the association between disease status (both diabetes and hypertension [*n* = 1283], diabetes only [*n* = 760], hypertension only [*n* = 9649] and no diabetes and hypertension [*n* = 27 226]) and cognitive function. Individuals with both diabetes and hypertension were set as the reference level. Model 2 = adjusted for age + sex + deprivation + ethnicity + educational attainment + body mass index + CVD + hypercholesterolaemia + smoking. Reaction time, pairs matching and trail‐making test B − A results were converted to the same direction of all other cognitive tests so that higher values indicate better cognitive performance and lower values indicate poorer cognitive performance compared with controls. CVD, cardiovascular disease

Next, we carried out the same analysis as above, instead using the cognitive test outcomes. The results for model 2 for the cognitive tests are presented in Figure 4 (model 1; the results can be found in Figure S6). The results from Figure 4 show that those with hypertension or no disease had faster reaction times and performed better on the symbol‐digit substitution test. Individuals without hypertension and diabetes also performed better on the matrix pattern and TMT. Interestingly, those with diabetes only performed worse on the verbal and numerical reasoning test compared with individuals with both diabetes and hypertension.

## 4 DISCUSSION

Using data from a large population‐based cohort, aged 40‐69 years, we aimed to investigate the complex inter‐relationships between diabetes, hypertension, neuroimaging measures and cognitive function. The main findings of our study are 2‐fold. Firstly, individuals with diabetes have overall poorer brain and cognitive health, as indexed by global, subcortical and WM regions and a range of cognitive tests. Secondly, individuals with both diagnosed diabetes and hypertension have poorer cognitive and brain health in comparison with those with hypertension only, and to a lesser extent those with only diabetes.

Individuals with diabetes had overall poorer brain health, as indexed by a range of volumetric brain variables. These associations were present in our fully adjusted models for all neuroimaging outcomes, except for gFA (a measure of WM integrity) and the amygdala (the region associated with emotional processing and fear‐related memories). These findings largely agree with previous studies of the association between diabetes and neuroimaging outcomes in the UKB.^6^, ^7^ However, some key distinctions between our study and theirs include some differences in modelling approaches (e.g. covariate adjustment), sample size (~10 000 vs. our sample of maximum ~40 000) and the definition of diabetes (self‐reported vs. use of a validated algorithm plus HbA1c measurements). Our study also investigated a much wider range of neuroimaging variables compared with at least one of the earlier studies,^7^ which only included hippocampal and WMH volumes.

Similarly, we observed that those with a diabetes diagnosis also had poorer performance on certain cognitive function tests, particularly for reaction time, verbal and numeric reasoning, trail‐making and symbol‐digit substitution tests. Our finding for reaction time is in line with two previous UKB studies^5^, ^7^ and the verbal numeric reasoning result is supported by one earlier study. More broadly, it has long been suggested that individuals with diabetes have slower reaction times. Almost 40 years ago, Subramanian and Chandrasekar^17^ showed that participants with diabetes had slower reaction times than age‐matched controls.

Taken together, our findings of an association between diabetes and certain neuroimaging outcomes, as well as specific cognitive tests, warrant discussion. This may indicate that diabetes has differential associations across brain regions and that, for example, given the lack of a relationship observed with amygdala volume, perhaps emotional processing is not impaired in diabetes. However, experimental studies in mice do suggest that diabetes dysregulates dopaminergic circuitry in the amygdala and that this negatively affects certain aspects of social behaviour.^18^ In relation to diabetes and gFA, it is also possible that when taking into account other important factors (fully adjusted models), diabetes may not have a large association with WM integrity in mid‐life. However, this should be interpreted with caution because, as mentioned above, an earlier UKB study suggested that diabetes does relate to poorer WM integrity.^6^ A recent systematic review in fact suggests that findings of the relationship between diabetes and WM integrity are equivocal, with some studies finding an association and others that do not.^19^

Perhaps our most novel and intriguing finding is the strong association between co‐morbid diabetes and hypertension and worse brain and cognitive health. Those with both conditions had worse overall brain health as measured by structural MRI and, as expected, individuals without either diabetes/hypertension had better brain health. There was no difference between those with only diabetes versus those with both diabetes and hypertension for total brain volume, ventricular CSF, thalamus, caudate, putamen, pallidum, hippocampus and amygdala. However, the largest difference was observed between those with only diabetes and those with both diseases in terms of total grey matter volume, a finding which agrees with a meta‐analysis of this association.^20^ Specifically, among the seven studies included in the meta‐analysis, individuals with diabetes had reduced grey matter compared with those without diabetes.

Having only hypertension versus both conditions was not associated with differences in the amygdala or gFA (WM integrity). These results suggest that hypertension alone is associated with better brain health across a breadth of brain regions in comparison with only having diagnosed diabetes. The largest difference we observed between hypertension and co‐morbid diabetes/hypertension was in the pallidum, a structure within the basal ganglia that plays an important part in reward processing.^21^ It is also important to note that when we examined WMH volume more closely (a marker of vascular brain damage) the size of the coefficient also suggested that hypertension related more strongly to this outcome in comparison with diabetes.

For cognitive function, the pattern of results was very similar to the brain imaging outcomes. Having only diabetes in comparison with both diabetes and hypertension conferred worse performance on the verbal and numerical reasoning task, but no other cognitive tests. Importantly, co‐morbid diabetes and hypertension was associated with poorer performance on the reaction time and symbol‐digit substitution (processing speed/executive function) tasks, in comparison with having only hypertension, with very similar effect sizes across both of these tests.

Finding that co‐morbid diabetes and hypertension associates with worse overall brain and cognitive health may be novel, but it is not surprising. Epidemiological research that is almost 2 decades old suggests that those with both conditions have an increased dementia risk.^22^ Our study indicates that co‐morbid diabetes and hypertension is strongly related to more objective, subclinical markers of dementia, as indexed by cognitive tests and structural MRI scans. From a clinical perspective, our findings suggest that perhaps there could be benefits for brain and cognitive health of using medication to treat both diabetes and hypertension in synergy. Substantial trial evidence shows that this has certainly been the case for microvascular and macrovascular complications.^23^ However, one major difference is that there is uncertainty about whether the associations that we observed in our study are causal in nature.

The strengths of our study include the large sample of participants with data on cardiovascular risk factors, cognitive tests and, in particular, structural brain MRI scans, the use of a validated algorithm and HbA1c levels to define prevalent diabetes,^24^ and that we analysed standardized cognitive tests that were administered at the UKB imaging visit in the UKB. However, we also acknowledge some important limitations: the UKB had a low response rate, which may result in selection bias,^25^ alongside some recent evidence that the imaging subsample has a ‘healthy’ bias over and above the baseline sample^26^; the use of self‐reported data for certain medical diagnoses, which could lead to misclassifications, and the use of observational data, which precludes causal conclusions. Furthermore, we did not adjust for measures of sedentary lifestyle (such as physical activity) and therefore our estimates may be overestimated. However, we adjusted for other important factors related to a sedentary lifestyle such as smoking and BMI. Future research could also try to disentangle whether associations between diabetes and brain/cognitive health vary depending on definition (clinical vs. self‐report vs. HbA1c).

In terms of the generalizability of our findings, we observed strong associations between diabetes and brain health even in comparatively healthy participants in the UKB, which could mean that these associations would be more pronounced in a sample with greater external validity. The fact that our sample is largely White European also restricts the generalizability to other ethnic groups.

In conclusion, diabetes is associated with worse overall global brain health, as measured by structural MRI, and not only those strongly related to dementia and cognitive decline (e.g. the hippocampus). This work also shows that those with neither hypertension nor diabetes, compared with those with either one, or both of these diseases, had better cognitive and brain health.

Our findings highlight that greater emphasis could be placed on trying to understand when these detectable differences in brain structure might occur, as this may prove to be crucial in terms of dementia progression in individuals with diabetes. Moreover, we show that individuals with both hypertension and diabetes have worse overall brain and cognitive health when compared with those with only one of these diseases. This finding is novel and could be a starting point for further studies that aim to support and justify cardiovascular risk factor management for brain and cognitive health more broadly. Overall, our research suggests that prevention of both diabetes and hypertension could delay structural brain changes, as well as potential future cognitive decline and dementia, but further clinical studies are needed.

## CONFLICT OF INTEREST

The authors declare no conflicts of interest.

## AUTHOR CONTRIBUTIONS

DN and VG both conceived and designed the study. DN carried out the analysis and both authors wrote the manuscript. Both authors accept full responsibility for the work and DN had access to the data, and both authors controlled the decision to publish.

### PEER REVIEW

The peer review history for this article is available at https://publons.com/publon/10.1111/dom.14658.

## Supporting information


**Appendix S1**. Supporting Information

## Data Availability

Data availability: UK Biobank is an open access resource available to verified researchers upon application (http://www.ukbiobank.ac.uk/). Analysis syntax is available on request. This research was conducted using the UK Biobank Resource under approved project 43309.
