# Supplementary material for: Understanding the inter‐relationships of type 2 diabetes and hypertension with brain and cognitive health: A UK Biobank study
Source: Diabetes Obes Metab. 2022 Feb 24;24(5):938–47. doi: 10.1111/dom.14658 (PMC9415107; doi:10.1111/dom.14658)
Supplement: Supplementary file 1 — Appendix S1. Supporting Information [file DOM-24-938-s001.docx]

**Supporting Information**

**Table S1. Self-reported health variables codes used for exclusion criteria on initial population**

| Condition | Code |
| --- | --- |
|  | *Self reported Illness (Field ID 20002)* |
| Dementia or Alzheimer’s disease | 1263 |
| Parkinson’s disease | 1262 |
| Chronic degenerative neurological | 1258 |
| Guillain-Barré syndrome | 1256 |
| Multiple Sclerosis | 1261 |
| Other demyelinating disease | 1397 |
| Brain cancer | 1032 |
| Brain haemorrhage | 1491 |
| Brain/intracranial abscess | 1245 |
| Cerebral aneurysm | 1425 |
| Cerebral palsy | 1433 |
| Encephalitis | 1246 |
| Epilepsy | 1264 |
| Head injury | 1266 |
| Infections of the nervous system | 1244 |
| Meningeal cancer | 1031 |
| Meningioma (benign) | 1659 |
| Meningitis | 1247 |
| Motor Neuron Disease | 1259 |
| Neurological injury/trauma | 1240 |
| Spina bifida | 1524 |
| Subdural haematoma | 1083 |
| Subarachnoid haemorrhage | 1086 |

**Table S2. UK Biobank Field codes for all variables used in manuscript**

| Variable | Code |
| --- | --- |
| ***Type 2 Diabetes variables*** |  |
| Self reported diagnosis | Field ID 20002, code 1223 |
| Self reported anti diabetic drug medications | Field ID 20002, code 1140874646, 1140874664, 1140874674, 1140874686, 1140874706, 1140874718, 1140874744, 1140884600, 1141189090, 1141171646, 1140868902, 1141168660, 1141152590, 1140883066, 1140874650, 1140874652, 1140874658, 1140874660, 1140874666, 1140874678, 1140874680, 1140874690, 1140874712, 1140874716, 1140874724, 1140874726, 1140874728, 1140874732, 1140874736, 1140874740, 1140874746, 1141177600, 1141171652, 1140868908, 1141168668, 1141157284 |
| First occurence | 130709 |
| HbA1c | 30750 |
| Diabetes diagnosed by a doctor | 2443 |
| Age of diabetes diagnosed | 2976 |
| ***Hypertension variables*** |  |
| Self reported diagnosis | Field ID 20002, Code 1065, 1072 |
| Age hypertension diagnosed | 2966 |
| BP medication use | Field IDs 6177, 6153 |
| ***Neuroimaging*** |  |
| Total Brain Volume (TBV) | 25010 |
| Total Grey Matter (TGM) | 25006 |
| Total White Matter (WM) | 25008 |
| White matter hyperintensities (WMH) | 25781 |
| Ventricular CSF | 25004 |
| Hippocampus (L+R) | 25019/20 |
| Thalamus (L+R) | 25011/12 |
| Caudate (L+R) | 25013/14 |
| Putamen (L+R) | 25015/16 |
| Pallidum (L+R) | 25017/18 |
| Amygdala (L+R) | 25021/22 |
| Accumbens (L+R) | 25023/24 |
| gFA (fractional anisotropy) | 25488-25514 |
| gMD (mean diffusivity) | 25515-25541 |
| ***Cognitive Tests*** |  |
| Symbol-Digit | 23324 |
| Matrix Reasoning | 6373 |
| Verbal and Numeric Reasoning | 20016 |
| Reaction Time | 20023 |
| Pairs Matching | 399 |
| TMT A | 6348 |
| TMT B | 6350 |
| Tower Rearranging | 21004 |
| ***Confounding Variables*** |  |
| Education | 6138 |
| Townsend deprivation index at recruitment | 189 |
| Smoking Status | 20116 |
| Gender | 31 |
| Age at Assessment | 21300 |
| Assessment Centre | 54 |
| BMI | 21001 |
| Ethnicity | 21000 |
| High Cholesterol | Field ID 20002, Code 1473 & Field IDs 6177, 6153 |
| Head size | 25000 |
| Scanner Position X | 25756 |
| Scanner Position Y | 25757 |
| Scanner Position Z | 25758 |
| Scanner Position | 25759 |

Field IDs obtained only for imaging visit apart from Ethnicity where baseline visit information was also used. For Hb1Ac values from baseline and second follow up were utilised.

**Definition of diabetes and hypertension phenotypes**

Based on the field variables defined in Table S2 we created phenotypes for diabetes and hypertension based on various related variables. Further information on the specific variables is specified below.

Self-report

We used information from self reported disease status and self reported medication use for both diabetes and hypertension reported at the imaging visit from touchscreen questionnaires and nurse interviews.

Clinical

For diabetes diagnosis, we used first occurrence variables provided by UK Biobank. This category contains data showing the 'first occurrence' of any code mapped to 3-character ICD-10.

The data-fields were generated by mapping:

1. Read code information in the Primary Care data (Category 3000),
2. ICD-9 and ICD-10 codes in the Hospital inpatient data (Category 2000),
3. ICD-10 codes in Death Register records (Field 40001, Field 40002), and
4. Self-reported medical condition codes (Field 20002) reported at the baseline or subsequent UK Biobank assessment centre visit

Biochemistry

HbA1c assays were performed using five Bio-Rad Variant II Turbo analysers, manufactured by Bio-Rad Laboratories, Inc. and employ a High Performance Liquid Chromatography (HPLC) method. A validation study ensured that the analysers underwent a multi-instrument comparison to ensure that they were in agreement4. More details are outlined in detail in the UK Biobank HbA1c protocol (Tierney A, Fry D, Almond R, et al. UK Biobank Biomarker Enhancement Project Companion Document to Accompany HbA1c Biomarker Data. 2018;1–8) .Available from: <https://biobank.ndph.ox.ac.uk/showcase/showcase/docs/serum_hb1ac.pdf>. For this work, any participant with an HbA1c greater than 48 mmol/mol was defined as undiagnosed diabetes and assigned to the diabetes group.

Below is a venn diagram which shows the overlap of the diabetes phenotypes using the different measures of self report, clinical and biochemistry variables for diabetic participants.


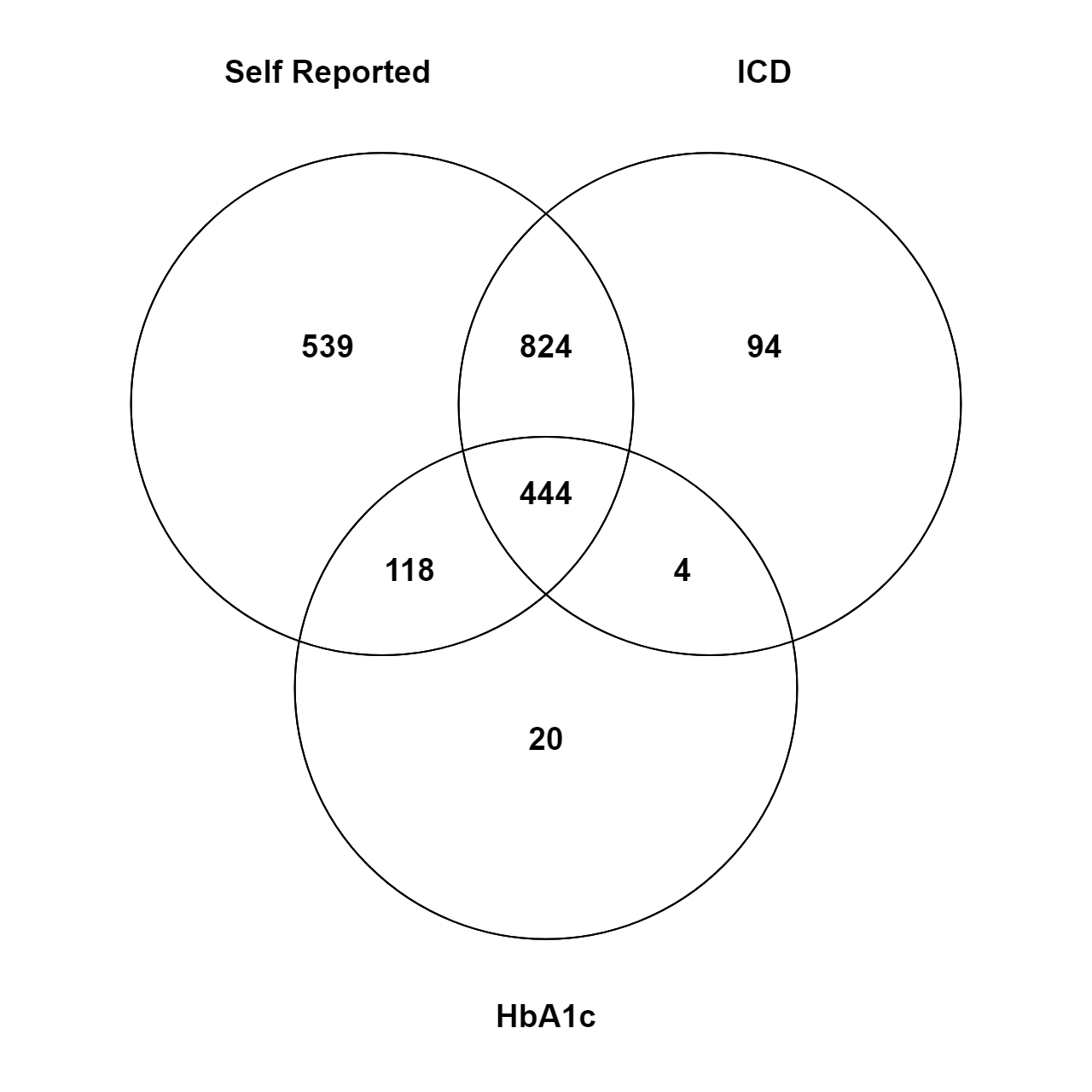


**Figure S1: Venn Diagram showing the overlap of participants with diabetes diagnosis using different diabetes phenotype variables in UK biobank. Self-report (diagnosis/medications) (n = 1925), biochemistry (n = 586) and clinical data (n = 1366) with 68% (n =1390) overlapping with two or more diabetes phenotypes.**

**Information on cognitive tests**

Further information of the cognitive function tests can be found on the UKBiobank website (<https://biobank.ndph.ox.ac.uk/showcase/label.cgi?id=100026>).

Verbal and Numerical Reasoning: A task with thirteen logic/reasoning-type questions and a two-minute time limit was labelled as ‘fluid intelligence’ in the UK Biobank protocol but is now referred to as ‘verbal-numerical reasoning’;<http://biobank.ctsu.ox.ac.uk/crystal/field.cgi?id=20016>). The maximum score is 13.

Pairs matching: A visual memory test was administered, labelled ‘pairs-matching’ (<http://biobank.ctsu.ox.ac.uk/crystal/label.cgi?id=100030>). Participants were asked to memorize the positions of six card pairs, and then match them from memory while making as few errors as possible. Scores on the pairs-matching test are for the number of errors that each participant made; therefore, higher scores reflect poorer cognitive function. The Pairs matching task had two versions: 3-pair and 6-pair. We used 6-pair version for this work. For the pairs matching, values over 30 were capped at 30, and only participants who completed the task were included.

Reaction time: Participants completed a timed test of symbol matching, similar to the common card game ‘Snap’. (<http://biobank.ctsu.ox.ac.uk/crystal/field.cgi?id=20023>). The score on this task was the mean response time in milliseconds across trials, which contained matching pairs.

From 2016 at the imaging visit additional validated cognitive tests were administered including Matrix Pattern, Symbol-Digit Substitution, tower rearranging and Trail-Making Tests (TMT) B and A.

Trail Making Test A and B: In this work, we used TMT B – A. Subtracting TMT A from TMT B removes the individual variance in speed of response and is considered a useful tool in clinical practice for dementia. Individuals who scored >250 s for TMT B were excluded as well as participants with a TMT B - TMT A score less than 0 and greater than 150 seconds were also excluded.

Matrix Pattern: The participant was presented with a series of matrix pattern blocks with an element missing and asked to select the element that best completed the pattern from a range of displayed choices. <https://biobank.ctsu.ox.ac.uk/crystal/label.cgi?id=501>

Symbol-Digit Substitution: The participant was presented with one grid linking symbols to single-digit integers and a second grid containing only the symbols. They were then asked to indicate the numbers attached to each of the symbols in the second grid using the first one as a key. <https://biobank.ctsu.ox.ac.uk/crystal/label.cgi?id=502>. The number of symbol digit matches made correctly were used.

Tower rearranging: The participant was presented with an illustration of three pegs (towers) on which three differently-coloured hoops had been placed. They were then asked to indicate how many moves it would take to rearrange the hoops into another specific position. The number of correct puzzles was reported. <https://biobank.ctsu.ox.ac.uk/crystal/label.cgi?id=503>


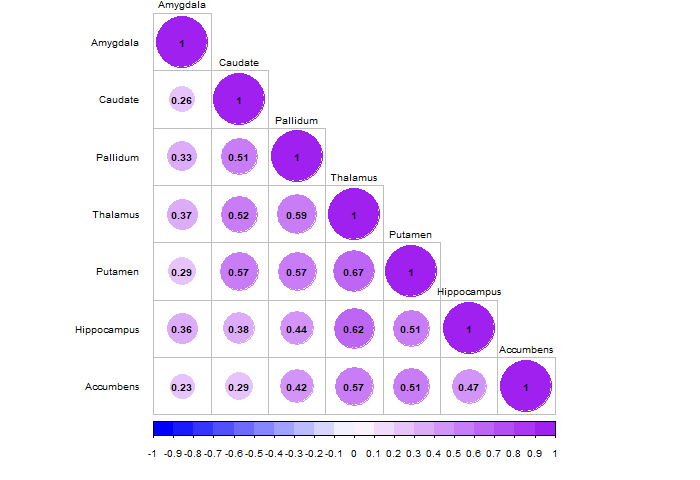


**Figure S2: Pearson's correlation matrix of subcortical regions brain measures. Higher values indicate higher correlation between brain subcortical regions**

**Table S3: Characteristics of UK Biobank participants at imaging visit included stratified by diabetes and hypertension diagnosis.**

| Description | No Disease (n=27226) | Hypertension (n = 9649) | Diabetes (n = 760) | Hypertension + diabetes (n = 1283) | N |
| --- | --- | --- | --- | --- | --- |
| Age years (mean (SD)) | 62.6 (7.52) | 66.1 (7.00) | 64.5 (7.49) | 66.6 (6.86) | 38918 |
| Gender (Male N (%)) | 11531 (42.4%) | 5499 (57.0%) | 464 (61.1%) | 844 (65.8%) | 38918 |
| Ethnicity (White N (%)) | 26422 (97.3%) | 9356 (97.2%) | 696 (91.8%) | 1179 (92.3%) | 38815 |
| Education (Degree N (%)) | 13797 (51.2%) | 4275 (44.7%) | 305 (40.6%) | 479 (37.8%) | 38525 |
| Townsend Deprivation (N (%)) |  |  |  |  |  |
| 1 | 5443 (20.0%) | 2011 (20.9%) | 116 (15.3%) | 219 (17.1%) | 38883 |
| 2 | 5549 (20.4%) | 1878 (19.5%) | 129 (17.0%) | 205 (16.0%) |  |
| 3 | 5403 (19.9%) | 1975 (20.5%) | 167 (22.0%) | 240 (18.7%) |  |
| 4 | 5456 (20.1%) | 1887 (19.6%) | 150 (19.7%) | 280 (21.9%) |  |
| 5 | 5348 (19.7%) | 1892 (19.6%) | 198 (26.1%) | 337 (26.3%) |  |
| Assessment Centre (N (%)) |  |  |  |  |  |
| Cheadle | 16926 (62.2%) | 6002 (62.2%) | 488 (64.2%) | 794 (61.9%) | 38918 |
| Reading | 3525 (12.9%) | 1245 (12.9%) | 90 (11.8%) | 162 (12.6%) |  |
| Newcastle | 6775 (24.9%) | 2402 (24.9%) | 182 (23.9%) | 327 (25.5%) |  |
| BMI Kg/m2 (mean (SD)) | 25.8 (4.04) | 27.8 (4.53) | 28.5 (4.97) | 30.5 (5.16) | 37626 |
| Smoking Status (N (%)) |  |  |  |  |  |
| Non Smoker | 17443 (64.7%) | 5655 (59.0%) | 417 (55.8%) | 675 (53.0%) | 38541 |
| Previous | 8527 (31.6%) | 3632 (37.9%) | 290 (38.8%) | 561 (44.1%) |  |
| Current | 973 (3.61%) | 291 (3.04%) | 40 (5.35%) | 37 (2.91%) |  |
| Hypercholesterolaemia (N (%)) | 3663 (13.5%) | 4402 (45.6%) | 416 (54.7%) | 1008 (78.6%) | 38918 |
| Hypertension (N (%)) | 0 (0.00%) | 9649 (100%) | 0 (0.00%) | 1283 (100%) | 38918 |
| Total Brain Volume mm3 (mean (SD)) | 1162722 (111172) | 1160787 (111180) | 1153345 (112068) | 1147126 (108711) | 38908 |
| Grey Matter mm3 (mean (SD)) | 617993 (55212) | 610659 (55783) | 606029 (56596) | 598138 (55738) | 38911 |
| WMH mm3 (median (IQR)) | 2394 (3276) | 3945 (5750) | 3448 (4644) | 4688 (7065) | 37168 |
| gFA units M (SD) | 0.05 (0.53) | -0.12 (0.59) | -0.01 (0.56) | -0.15 (0.58) | 34718 |
| gMD units M (SD) | -0.05 (0.43) | 0.13 (0.50) | 0.02 (0.46) | 0.17 (0.51) | 34718 |
| Ventricular CSF mm3 (mean (SD)) | 34368 (15119) | 39926 (17096) | 39642 (16811) | 43270 (18088) | 38721 |
| Hippocampus mm3 (mean (SD)) | 3857 (430) | 3804 (438) | 3779 (453) | 3740 (441) | 38876 |
| Accumbens mm3 (mean (SD)) | 450 (104) | 423 (104) | 420 (104) | 398 (102) | 38902 |
| Amygdala mm3 (mean (SD)) | 1244 (215) | 1249 (218) | 1244 (220) | 1249 (216) | 38896 |
| Pallidum mm3 (mean (SD)) | 1781 (219) | 1771 (228) | 1738 (213) | 1721 (244) | 38835 |
| Putamen mm3 (mean (SD)) | 4809 (564) | 4781 (583) | 4736 (561) | 4669 (575) | 38872 |
| Caudate mm3 (mean (SD)) | 3468 (416) | 3486 (426) | 3417 (417) | 3423 (421) | 38870 |
| Thalamus mm3 (mean (SD)) | 7690 (729) | 7602 (717) | 7516 (721) | 7434 (731) | 38851 |
| Pairs Matching - incorrect matches (mean (SD)) | 3.58 (2.81) | 3.87 (3.00) | 3.75 (3.28) | 3.86 (2.97) | 35898 |
| Verbal and Numerical Reasoning – Correct answers (mean (SD)) | 6.70 (2.05) | 6.53 (2.06) | 6.10 (2.06) | 6.37 (2.17) | 35837 |
| Reaction Time in seconds (median (IQR)) | 570 (125) | 585 (133) | 589 (120) | 597 (140) | 36323 |
| Trail-Making Test B – A in seconds (median (IQR)) | 285 (190) | 312 (216) | 323 (224) | 330 (245) | 24402 |
| Matrix Reasoning – Correct answers (mean (SD)) | 8.10 (2.10) | 7.76 (2.16) | 7.67 (2.31) | 7.45 (2.27) | 25286 |
| Symbol-Digit Substitution – Correct answers (mean (SD)) | 19.5 (5.17) | 18.0 (5.25) | 17.8 (5.26) | 16.9 (5.39) | 25320 |
| Tower Rearranging – Correct answers (mean (SD)) | 10.0 (3.22) | 9.64 (3.21) | 9.56 (3.32) | 9.53 (3.40) | 25074 |

Townsend Deprivation split into quintiles where 5 is most deprived, IQR:Interquartile range. For the brain measures larger values for white matter hyperintensities (WMH), ventricular CSF and g Mean Diffusivity (gMD) indicate poorer brain health whereas for all other brain measures smaller values indicate poorer brain health. For the cognitive tests lower values indicate poorer cognition for verbal and numerical reasoning, matrix reasoning, symbol digit substitution and tower arranging and for pairs matching, reaction time and trail-making test B-A higher values indicate poorer cognition.

**Table S4: Table showing the standardized betas for diabetes and hypertension as individuals covariates from the association between diabetes (n=2043 cases, n=36875 controls) and neuroimaging outcomes.** Model 2 = Model 1 + BMI + CVD + hypercholesterolaemia + hypertension + smoking. WMH, Vascular CSF, gMD results were converted to the same direction of all other brain measures so that higher values indicate better brain health compared to reference level, for ease of comparisons.

| **Description** | **Covariate** | **Standardized Beta** | **Lower 95% CI** | **Upper 95% CI** |
| --- | --- | --- | --- | --- |
| Total Brain Volume (n =37045) | Diabetes | -0.077 | -0.097 | -0.057 |
|  | Hypertension | -0.021 | -0.032 | -0.011 |
| Total Grey Volume (n =37048) | Diabetes | -0.142 | -0.164 | -0.119 |
|  | Hypertension | -0.054 | -0.066 | -0.043 |
| WMH (n =35370) | Diabetes | 0.114 | 0.073 | 0.155 |
|  | Hypertension | 0.218 | 0.197 | 0.240 |
| Ventricular CSF (n =36868) | Diabetes | 0.168 | 0.130 | 0.206 |
|  | Hypertension | 0.072 | 0.052 | 0.092 |
| Thalamus (n =36988) | Diabetes | -0.157 | -0.190 | -0.125 |
|  | Hypertension | -0.034 | -0.051 | -0.017 |
| Caudate (n =37007) | Diabetes | -0.096 | -0.136 | -0.057 |
|  | Hypertension | 0.040 | 0.020 | 0.061 |
| Putamen (n =37007) | Diabetes | -0.145 | -0.181 | -0.109 |
|  | Hypertension | 0.002 | -0.017 | 0.021 |
| Pallidum (n =36976) | Diabetes | -0.179 | -0.220 | -0.137 |
|  | Hypertension | -0.026 | -0.047 | -0.004 |
| Hippocampus (n =37011) | Diabetes | -0.103 | -0.144 | -0.061 |
|  | Hypertension | -0.034 | -0.056 | -0.012 |
| Amygdala (n =37031) | Diabetes | -0.017 | -0.061 | 0.027 |
|  | Hypertension | -0.048 | -0.071 | -0.025 |
| Accumbens (n =37037) | Diabetes | -0.153 | -0.194 | -0.111 |
|  | Hypertension | -0.090 | -0.111 | -0.068 |
| gFA (n =34187) | Diabetes | -0.023 | -0.050 | 0.003 |
|  | Hypertension | -0.122 | -0.136 | -0.108 |
| gMD (n =34187) | Diabetes | 0.050 | 0.029 | 0.071 |
|  | Hypertension | 0.115 | 0.104 | 0.126 |

**
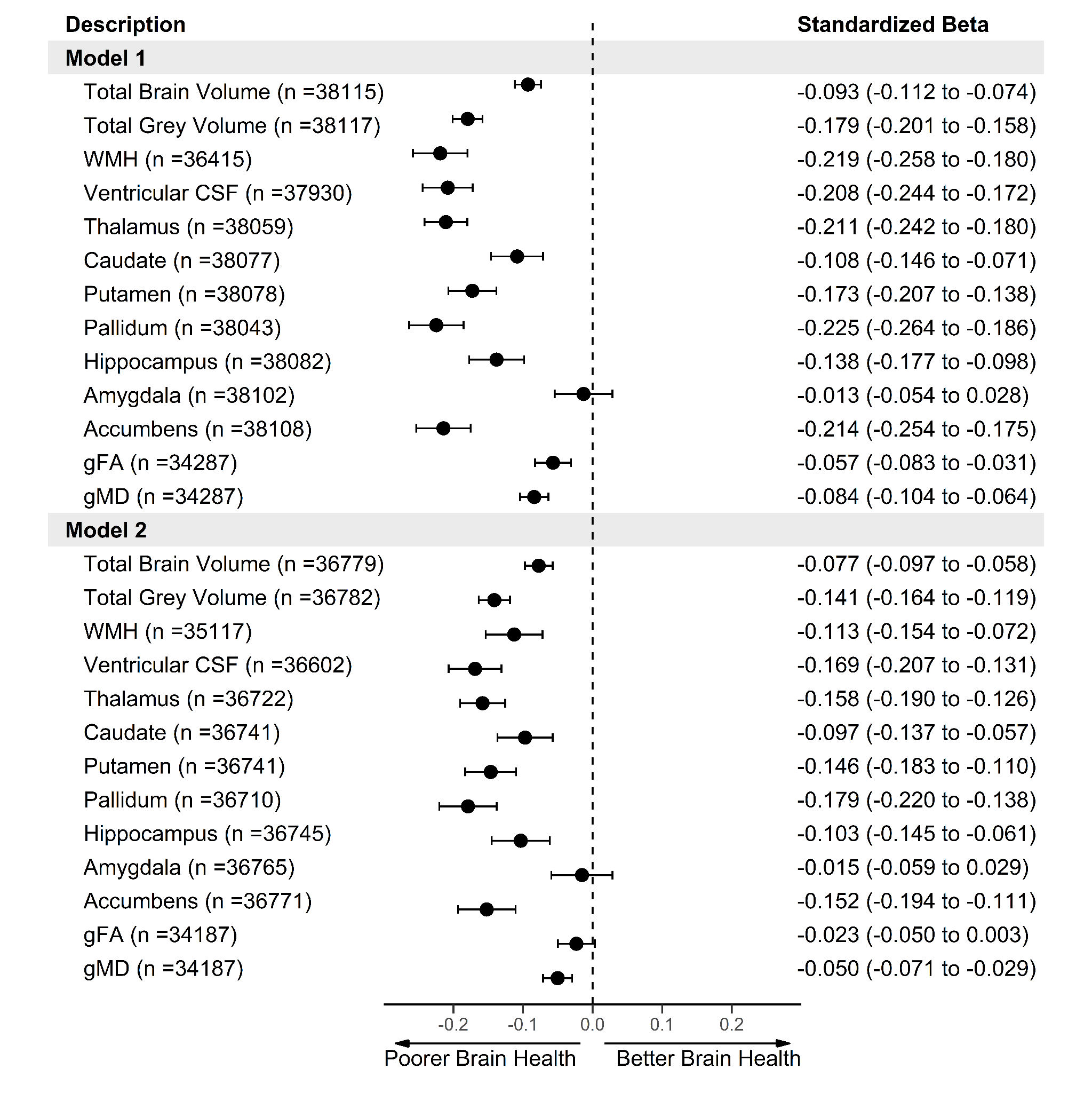
Figure S3: Forest plot of the association between diabetes (n=2041 cases, n=36610 controls) and neuroimaging outcomes after removal of participants with low BMI (< 18.5kg/m2)(n = 267).** Model 1 = adjusted for age + sex + deprivation + ethnicity + educational attainment + head size + scanner position variables. Model 2 = Model 1 + BMI + CVD + hypercholesterolaemia + hypertension + smoking. WMH, Vascular CSF, gMD results were converted to the same direction of all other brain measures so that higher values indicate better brain health compared to reference level, for ease of comparisons.

**Table S5: Table showing the standardized betas for diabetes and hypertension as individuals covariates from the association between diabetes (n=2043 cases, n=36875 controls) and cognitive function.** Model 2 = Model 1 + BMI + CVD + hypercholesterolaemia + hypertension + smoking. Reaction time, pairs matching and Trail making test B-A results were converted to the same direction of all other cognitive tests so that higher values indicate better cognitive performance and lower values indicate poorer cognitive performance compared to controls.

| **Description** | **Covariate** | **Standardized Beta** | **Lower 95% CI** | **Upper 95% CI** |
| --- | --- | --- | --- | --- |
| Reaction Time (n =34815) | Diabetes | 0.111 | 0.064 | 0.158 |
|  | Hypertension | 0.014 | -0.010 | 0.039 |
| Verbal & Numeric Reasoning (n =34351) | Diabetes | -0.067 | -0.115 | -0.019 |
|  | Hypertension | -0.001 | -0.026 | 0.024 |
| Pairs Matching (n =34401) | Diabetes | -0.014 | -0.064 | 0.036 |
|  | Hypertension | 0.036 | 0.011 | 0.062 |
| Trail Making Test B - A (n =23099) | Diabetes | 0.081 | 0.022 | 0.140 |
|  | Hypertension | 0.022 | -0.008 | 0.053 |
| Matrix Pattern (n =23915) | Diabetes | -0.060 | -0.117 | -0.003 |
|  | Hypertension | -0.037 | -0.066 | -0.007 |
| Symbol Digit Substitution (n =23943) | Diabetes | -0.133 | -0.187 | -0.079 |
|  | Hypertension | -0.049 | -0.077 | -0.021 |
| Tower Rearranging (n =23720) | Diabetes | -0.022 | -0.081 | 0.038 |
|  | Hypertension | -0.036 | -0.066 | -0.005 |


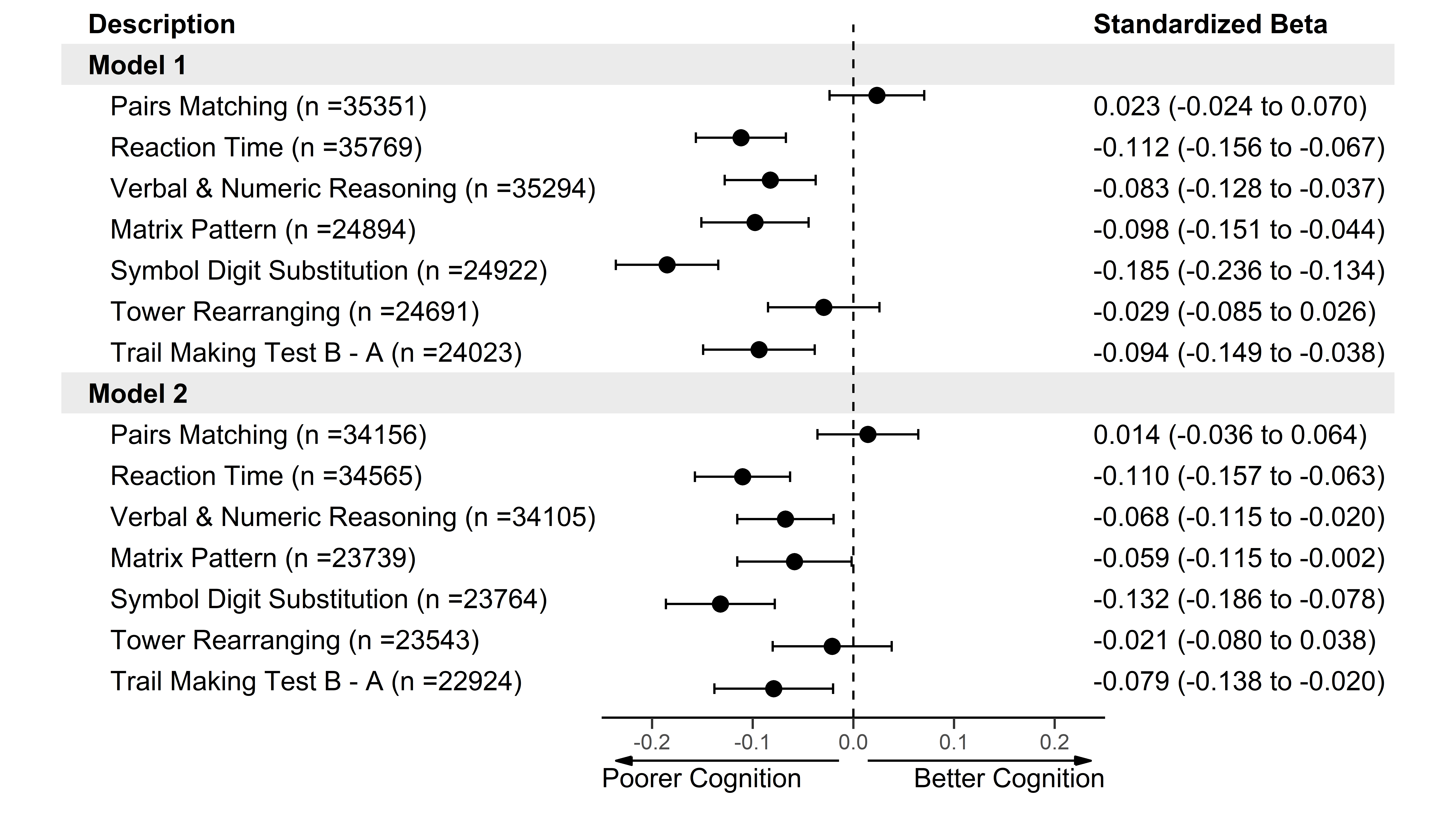


**Figure S4: Forest plot of the association between diabetes (n=2041 cases, n=36610 controls) and cognitive function after removal of participants with low BMI (< 18.5kg/m2)(n = 267).** Model 1 = adjusted for age + sex + deprivation + ethnicity + educational attainment. Model 2 = Model 1 + BMI + CVD + hypercholesterolaemia + hypertension + smoking. Reaction time, pairs matching and Trail making test B-A results were converted to the same direction of all other cognitive tests so that higher values indicate better cognitive performance and lower values indicate poorer cognitive performance compared to controls.


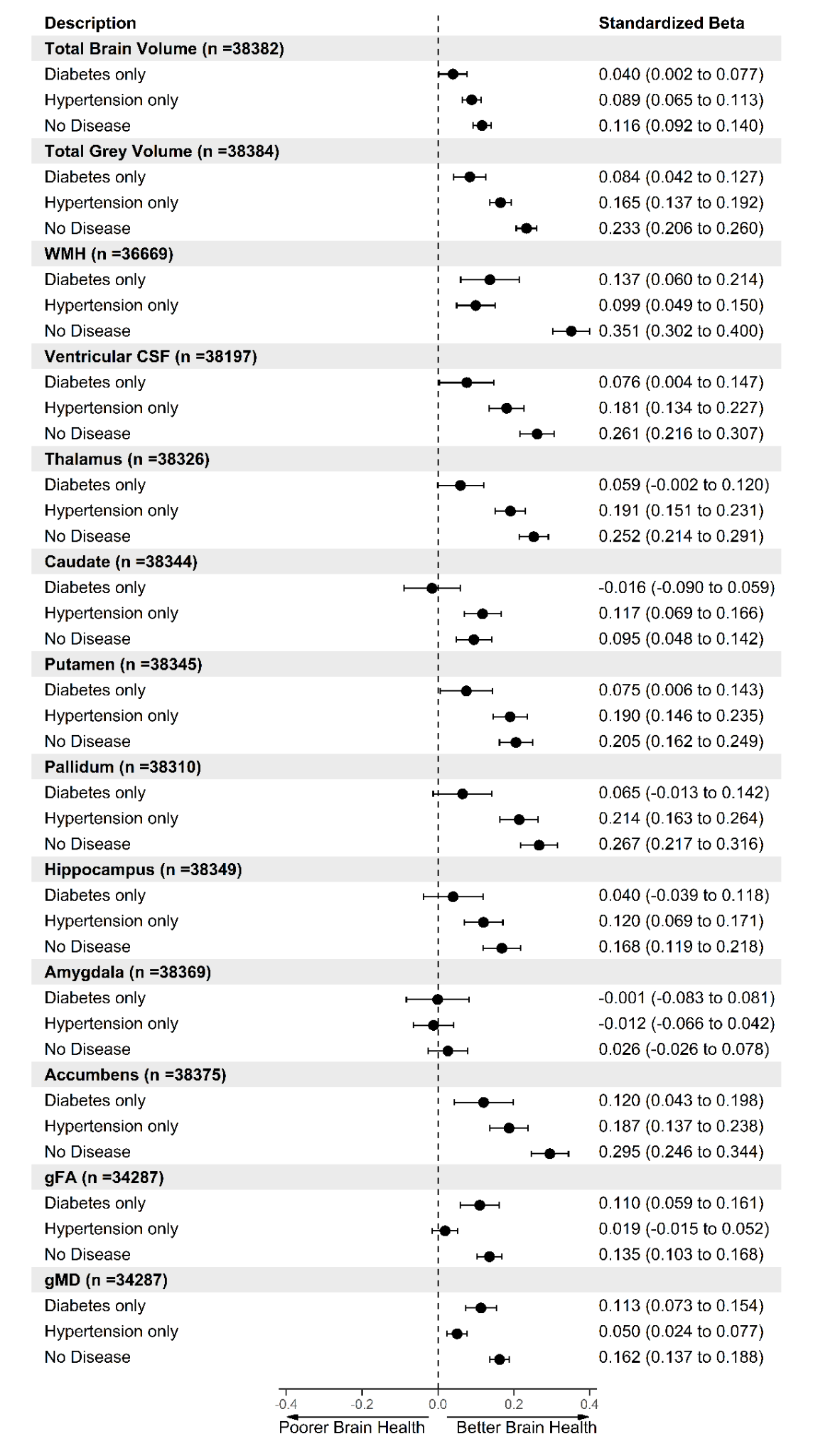


**Figure S5: Forest plot of the association between disease status [both diabetes and hypertension (n = 1283), diabetes only (n = 760), hypertension only (n = 9649) and no diabetes and hypertension (n = 27226)] and neuroimaging outcomes.** Individuals with both diabetes and hypertension were set as the reference level. Model 1 = adjusted for age + sex + deprivation + ethnicity + educational attainment + head size + scanner position variables. WMH, Vascular CSF, gMD results were converted to the same direction of all other brain measures so that higher values indicate better brain health compared to reference level for ease of comparisons.





**Figure S6: Forest plot showing the association of different cognitive measures between individuals with both diabetes and hypertension (n = 1283), diabetes only (n = 760), hypertension only (n = 9649) and no diabetes and hypertension (n = 27226).** Individuals with both diabetes and hypertension were set as the reference level. Model 1 = adjusted for age + sex + deprivation + ethnicity + educational attainment. Reaction time, pairs matching and Trail making test B-A results were converted to the same direction of all other cognitive tests so that higher values indicate better cognitive performance and lower values indicate poorer cognitive performance compared to controls.
